# Comparison of TPF and PF induction chemotherapy combined with cisplatin concurrent chemoradiotherapy for locoregionally advanced nasopharyngeal carcinoma: A systematic review and meta-analysis

**DOI:** 10.1097/MD.0000000000041278

**Published:** 2025-01-17

**Authors:** Haiwen Li, Qibiao Wu, Haiqing Luo, Jiayuan Wu, Wenmei Su, Lili Yu

**Affiliations:** a Faculty of Chinese Medicine, Macau University of Science and Technology, Macau, P.R. China; b Department of Head and Neck Oncology, Affiliated Hospital of Guangdong Medical University, Zhanjiang, Guangdong, P.R. China; c The State Key Laboratory for Quality Research in Chinese Medicines of the Macau University of Science and Technology, Macau, P.R. China; d Guangdong-Hong Kong-Macao Joint Laboratory for Contaminants Exposure and Health, Guangdong University of Technology, Guangzhou, Guangdong, P.R. China; e Zhuhai MUST Science and Technology Research Institute, Zhuhai, Guangdong, P.R. China; f Clinical Research Service Center, Affiliated Hospital of Guangdong Medical University, Zhanjiang, Guangdong, P.R. China; g Department of Pulmonary Oncology, Affiliated Hospital of Guangdong Medical University, Zhanjiang, Guangdong, P.R. China.

**Keywords:** concurrent chemoradiotherapy, efficacy, induction chemotherapy, meta-analysis, nasopharyngeal carcinoma

## Abstract

**Background:**

The standard of care for locoregionally advanced nasopharyngeal carcinoma (LA-NPC) is induction chemotherapy (ICT) followed by concurrent chemoradiation (CCRT). However, the ideal ICT regimen for LA-NPC remains unclear. We conducted a meta-analysis to evaluate the survival outcomes, responses, and incidences of toxicities between taxane, cisplatin and fluorouracil (TPF) and cisplatin and fluorouracil (PF) ICT regimens plus CCRT in LA-NPC.

**Methods:**

A systematic review and meta-analysis of the literature was conducted to compare the efficacy and safety of TPF versus PF followed by CCRT with cisplatin every 3 weeks or weekly cisplatin and intensity-modulated radiotherapy in LA-NPC.

**Results:**

Three studies with 2482 patients met the inclusion criteria. ICT with the TPF regimen plus CCRT resulted in a significantly improved progression-free survival (hazard ratios [HR] 0.84 [95% CI 0.73–0.96], *P* = .01), overall survival (HR 0.83 [95% CI 0.71–0.97], *P* = .02), and 3-year locoregional recurrence-free survival (risk ratios [RR] 1.03 [95% CI 1.01–1.06], *P* = .009) compared with the PF regimen plus CCRT in LA-NPC. However, distant metastasis-free survival was not statistically significant (*P* = .07). The incidence of grade 3 or 4 neutropenia (RR 2.08 [95% CI 1.84–2.36]) and diarrhea (RR 1.94 [95% CI 1.07–3.52]) during ICT was higher in the TPF group than in the PF group.

**Conclusions:**

In terms of progression-free survival, overall survival, locoregional recurrence-free survival, in the era of intensity-modulated radiotherapy, the TPF plus CCRT with cisplatin is superior to PF plus CCRT with cisplatin in LA-NPC. Thus, the TPF plus CCRT regimen appears to be a reasonable treatment option, and further confirmation by prospective studies is needed.

## 1. Introduction

A unique head and neck tumor, nasopharyngeal carcinoma (NPC) is relatively endemic in southern China, southeast Asia, and northern Africa.^[1]^ Radiotherapy is the primary curative treatment for NPC. Early-stage NPC (I–II) is mainly treated with only radiotherapy, while LA-NPC (III–IVa) requires chemotherapy combined with radiotherapy, and the standard treatment for locoregionally advanced nasopharyngeal carcinoma (LA-NPC) is induction chemotherapy (ICT) with platinum-based chemotherapy followed by concurrent radiochemotherapy.^[2,3]^

Currently, intensity-modulated radiotherapy (IMRT) is widely used. IMRT has significantly improved local and regional control, and the prominent failure pattern is distant metastasis.^[4]^ Luo et al^[5]^ found that high-risk LA-NPC patients benefited from concurrent chemoradiation (CCRT) after ICT regarding disease-free survival, overall survival (OS), while low- and intermediate-risk patients did not. Indeed, many studies indicated the need for further intensification of systemic treatment to reduce the rate of distant failure and improve survival outcomes. However, owing to the poor compliance to adjuvant chemotherapy plus CCRT, ICT plus CCRT might be an attractive option for treatment intensification.^[6–8]^ ICT before CCRT was advantageous for eliminating clinically undetectable micrometastatic lesions and eliminating more local and potentially distant metastasis tumor cells. In addition, by shrinking the primary tumor, ICT could reduce the target volume and dose for the tumor regression field, resulting in reduced radiation toxicity and improved quality of life scores.^[9–11]^ Indeed, a number of phase II/III clinical trials have demonstrated that ICT followed by CCRT can improve survival outcomes.^[12,13]^ Sun et al^[14]^ reported that docetaxel, cisplatin, and fluorouracil (TPF) followed by the CCRT arm significantly improved 5year OS (hazard ratio [HR] 0.65 [95% CI 0.43–0.98]), failurefree survival (HR 0.65 [0.43–0.98]), and distant failure-free survival (DMFS, HR 0.60 [0.38–0.95]) in LA-NPC (excluding N0 disease) compared with the CCRT arm. Similarly, Cao et al^[15]^ found that 3year diseasefree survival was significantly improved in the cisplatin and fluorouracil (PF) followed by CCRT arm (82.0%, 95% CI = 0.77–0.87) compared with the CCRT alone arm (74.1%, 95% CI = 0.68–0.80, *P* = .028) in LA-NPC. Zhang et al^[16]^ revealed that the 3-year recurrence-free survival was 85.3% in the gemcitabine and cisplatin (GP) followed by the CCRT group and 76.5% in the CCRT alone group (HR, 0.51; 95% CI 0.34–0.77; *P* = .001). The 3-year OS was 94.6% in the GP plus CCRT group and 90.3% in the CCRT group (HR, 0.43; 95% CI, 0.24–0.77) in LA-NPC. A randomized phase II trial^[12]^ reported that the 3-year OS for TP plus CCRT versus CCRT alone arm was 94.1% and 67.7% (HR, 0.24; 95% CI, 0.078–0.73; *P* = .012), and the 3-year progression-free survival (PFS) for TP plus CCRT versus CCRT alone arm was 88.2% and 59.5% (HR 0.49; 95% CI 0.20–1.19; *P* = .12). Despite the fact that ICT plus CCRT has been validated as an effective treatment regimen, the most effective ICT regimen with fewer side effects remains unknown.

Earlier meta-analyses or network meta-analyses integrated information from all relevant studies, including different ICT regimens plus CCRT versus CCRT alone, to explore the optimal ICT regimens for clinical practice in LA-NPC. However, those studies included multiple radiotherapy techniques: two-dimensional radiotherapy, three-dimensional radiotherapy, and IMRT. In addition, different concurrent chemotherapy regimens included cisplatin, carboplatin, nedaplatin, and lobaplatin. Furthermore, a direct “head-to-head” comparison of different ICT regimens plus CCRT randomized clinical trials (RCTs) was not included. Those factors leading to the optimal ICT regimen for LA-NPC are still controversial.^[17–19]^ To date, few “head-to-head” RCTs have directly compared the different ICT regimens plus CCRT in terms of their effectiveness and safety profiles.^[20–22]^ Consequently, ICT benefits and regimens need to be updated.

There are many ICT regimens. In clinical practice, TPF, PF, TP, and GP are the most common ICT regimens for LA-NPC.^[23]^ Currently, 3 clinical trials directly comparing TPF plus CCRT to PF plus CCRT on the efficacy and safety profiles are reported. Therefore, an updated meta-analysis is warranted. An open-label, randomized, noninferiority trial was conducted in Chinese patients with LA-NPC to compare PFS, tolerance and compliance to TPF plus CCRT versus PF plus CCRT. The results showed that in the TPF plus CCRT arm, a PFS benefit was observed, but the difference was not statistically significant (84.5% vs 77.9%, *P* = .380) and the PF plus CCRT arm has substantially higher tolerance and compliance rates than the TPF plus CCRT arm.^[24]^ Similarly, Ma et al conducted an RCT in LA-NPC to compare the survival outcomes and tolerance of ICT with TPF followed by CCRT versus PF followed by CCRT. The results showed that the 5-year PFS (84.48% vs 82.75%, *P* = .458) and the 5-year OS (87.06% vs 85.34%, *P* = .274) were not significantly different between the TPF followed by CCRT group and the PF followed by CCRT group, and the incidences of adverse events (AEs) in the TPF followed by CCRT group were significantly higher than those in the PF followed by CCRT group (*P* < .001).^[25]^ However, another phase III randomized trial^[26]^ showed inconsistent results regarding efficacy and safety for patients with stage IVa to IVb NPC.

As the results are inconsistent, in order to explore the ideal ICT regimen and provide potential suggestions for clinical practice, a systematic review and meta-analysis was performed to compare the effectiveness and safety profiles of TPF and PF ICT followed by concurrent chemotherapy with cisplatin in IMRT era in patients with LA-NPC.

## 2. Materials and methods

### 2.1. Literature search strategy

This systematic review and meta-analysis was registered with PROSPERO (CRD42022382653) and was performed according to the Preferred Reporting Items for Systematic Reviews and Meta-analyses (PRISMA) guidelines.^[27]^ The published literature from electronic databases, including PubMed, Embase, Cochrane Library, Web of Science, Wanfang data and China National Knowledge Infrastructure (CNKI) database, were searched up to April 2023. The search terms were as follows: (“nasopharyngeal carcinoma” or “nasopharyngeal neoplasms” or “nasopharyngeal cancer”) and (“induction chemotherapy” or “neoadjuvant chemotherapy”) and (“concurrent chemoradiotherapy” or “concomitant chemoradiotherapy” or “synchronous chemoradiotherapy”) and (“randomized controlled trial” or “RCT” or “randomized” or “clinical trial”). Only studies in English and Chinese were included. To ensure a comprehensive review, registered clinical trials and published meeting proceedings or abstracts from conferences were consulted.

### 2.2. Literature selection criteria

All included studies met the following inclusion criteria: (a) [P] patients were diagnosed with stage III–IVa LA-NPC according to the American Joint Committee on Cancer or the International Union Against Cancer 5th to 8th editions staging criteria; (b) patients were over 18 years old; (c) [I, C] treatment measures compared different ICT schemes between TPF (paclitaxel/docetaxel, cisplatin, fluorouracil) and PF (cisplatin and fluorouracil) followed by CCRT; (d) [I, C] cisplatin monotherapy and IMRT was used during the radiotherapy period; (e) the type of trial was prospective RCT and retrospective propensity score-matched (PSM) trial; and (d) [O] Outcomes: data on survival outcomes, including PFS, or OS, or locoregional recurrence-free survival (LRRFS), or DMFS, and treatment-related AEs were available.

The exclusion criteria were as follows. (a) study results from the same research institution were excluded; (b) studies with ICT + CCRT versus CCRT; (c) single-arm studies with ICT + CCRT; (d) studies with adjuvant chemotherapy and dosing regimens. (e) Radiotherapy concurrent with nedaplatin, carboplatin, lobaplatin; (f) recurrent and metastatic NPC; (g) 3D-CRT or two-dimensional radiotherapy; (h) data were not available for analysis.

### 2.3. Quality assessments and data extraction

Cochrane Collaboration’s risk of bias tool was used to assess the quality of the included RCTs,^[28]^ and the overall risk of bias for each RCT was evaluated and rated as follows: low risk (−), unclear risk (?), and high risk (+). In addition, the Newcastle–Ottawa Scale ^[29]^ was used to assess the risk of bias in non-randomized controlled study. In brief, a maximum of 9 points was assigned to each study: 4 points for selection, 2 points for comparability, and 3 points for outcomes.

Two researchers screened studies independently and reviewed them with a third author. Data were collected from RCTs and PSMs, including author names, publication date, sample size, intervention and control regimens, chemotherapy and radiotherapy, median follow-up time, details of survival outcomes (OS, PFS, DMFS, and LRRFS), AEs ≥ G3 during ICT. The primary outcomes were PFS and OS. The secondary outcomes were DMFS, LRRFS, and AEs. AEs included hematological toxicity and nonhematological toxicity during ICT for analysis.

### 2.4. Statistical analysis

By synthesizing the results of all direct comparisons, we evaluated the efficacy and safety of different ICTs for patients with LA-NPC. Review Manager software (RevMan5.4, Cochrane Collaboration, Oxford, UK) was used in the following statistical analysis. Time-to-event data (OS and PFS) were expressed as HRs with 95% CIs. Risk ratios (RRs) with 95% CIs were used as summary statistics for LRRFS, DMFS, and AEs. CI *P* < .05 or 95% CI excluding 1 was defined as a statistically significant difference. Statistical heterogeneity was assessed via I^2^ and Cochran Q test values, when the *P* value of the Cochrane Q test was < .10 or the I^2^ > 50% was considered significant for heterogeneity. If *P* > .10 and I^2^ < 50%, a fixed-effect model was used to analysis. Otherwise, a random-effects model was performed.^[30]^

## 3. Results

### 3.1. Eligible studies and characteristics

**Figure 1.**
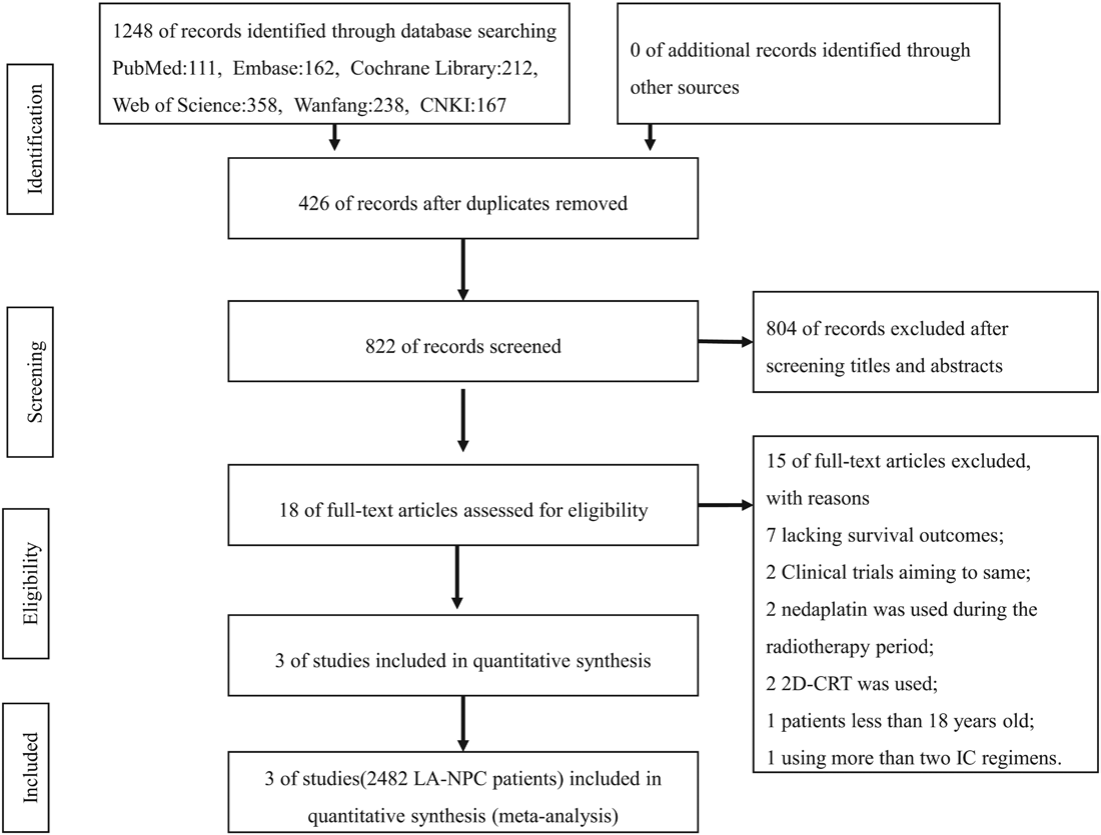
PRISMA flow diagram of the study selection process.

In the initial database search, 1248 studies were identified and after excluding 426 duplicates and 822 records were discarded by screening titles and abstracts. Of screening and eligibility assessment in full text, 2 prospective clinical trials and 1 retrospective propensity score-matched study involving 2482 patients were selected for subsequent statistical analysis.^[24,25,31–33]^ Among them, 3 clinical trials aim to be the same trial: 1 is interim analysis,^[32]^ and the other is update long-term result analysis.^[31]^ The PRISMA flow chart is presented in Figure 1. The main characteristics of the included studies investigating different ICT regimens followed by CCRT with cisplatin and IMRT in LA-NPC are summarized in Table 1. The trials were published between 2016 and 2019. All patients in the 3 trials were from China, and all studies used the UICC/AJCC 7th or 8th edition of staging. Two prospective trials were conducted with 3 cycles of ICT, and the ICT regimen was docetaxel plus cisplatin plus 5-fluorouracil,^[24,25]^ while a retrospective propensity score-matched study was performed with 2 to 4 cycles of ICT.^[33]^ The ICT scheme of all control groups was cisplatin plus 5-fluorouracil. During CCRT, all 3 trials were treated with triweekly or weekly cisplatin combined with IMRT. The dose of RT to the primary site was not <66 Gy in all the trials. Noticeably, the doses of ICT regimens and cisplatin with CCRT used were not the same. All the trials included patients more than 18 years old.

**Table 1:** Principal characteristics of included clinical trials with TPF or PF plus CCRT in LA-NPC.

Author	Year	Study type	Study center design	No. patients	Stage	ICT regimen	CCRT regimen	IMRT	Median follow-up (months)	Types of outcome measures	NOS score
Ma	2016	RCT	Single center	116	III–IVa (T1-4N 2-3, UICC 7th)	Docetaxel (60 mg/m^2^) + cisplatin (60 mg/m^2^) + fluorouracil (750 mg/m^2^/day) for 120 h, Q3W ×3 cycles	Cisplatin (80 mg/m^2^), 3-weeklys×2 cycles	PGTnx: 6 8.1 Gy/30 F, 5 f/W, over 6 weeks	48	PFS, OS, ORR	
				116		Cisplatin (80 mg/m^2^) + fluorouracil (750 mg/m^2^/day) for 120 h, Q3W ×3 cycles					
Jin	2019	RCT	Multi-center	138	III–IVa (AJCC 7th)	Docetaxel (75 mg/m^2^) + cisplatin (75 mg/m^2^) + fluorouracil (600 mg/m^2^/day) for 96 h, Q3W ×3 cycles	Cisplatin (80 mg/m^2^), 3-weeklys×2 cycles	PGTVnx: 69–70.4 Gy/30–32 F, 5 f/W, over 6 weeks	36 (24–48), Long-term outcomes update:99 m	PFS, OS, DMFS, LRRFS, ORR	
				138		Cisplatin (100 mg/m^2^) + fluorouracil (800 mg/m^2^/day) for120 h, Q3W ×3 cycles					
Peng	2018	Cohort: propensity score-matched (retrospective)	Single center	987	III–IVa (AJCC 8th)	Docetaxel + cisplatin + fluorouracil (specific doses were not described)	Cisplatin (80–100 mg/m^2^), 3-weeklys×3 cycles or weekly cisplatin (30–40 mg/m^2^).	PGTVnx: 66–70 Gy/30–33 F, 5 f/W	46.1 (0.57–91.5) m	PFS, OS, DMFS, LRRFS	8
				987		Cisplatin + fluorouracil (specific doses were not described)					

CCRT = concurrent chemoradiation, ICT = induction chemotherapy, IMRT = intensity-modulated radiotherapy, LA-NPC = locoregionally advanced nasopharyngeal carcinoma, LRRFS = locoregional recurrence-free survival, NOS = Newcastle–Ottawa Scale, OS = overall survival, PF = cisplatin and fluorouracil, PFS = progression-free survival, RCTs = randomized clinical trials, TPF = taxane, cisplatin and fluorouracil.

### 3.2. Quality assessment

Two RCTs were evaluated by the Cochrane Risk of Bias tool and the non-randomized controlled study was assessed by the Newcastle–Ottawa Scale to assess study quality. Allocation concealment was not reported in 2 RCTs. Ma’s study without complete outcome data. The particularity of ICT makes it difficult for researchers and participants to receive double-blind treatment. Since this has little impact on outcomes, the performance bias of all RCTs was evaluated as low risk. The non-randomized controlled study was high quality study with a score of 8.

### 3.3. PFS and OS

**Figure 2.**
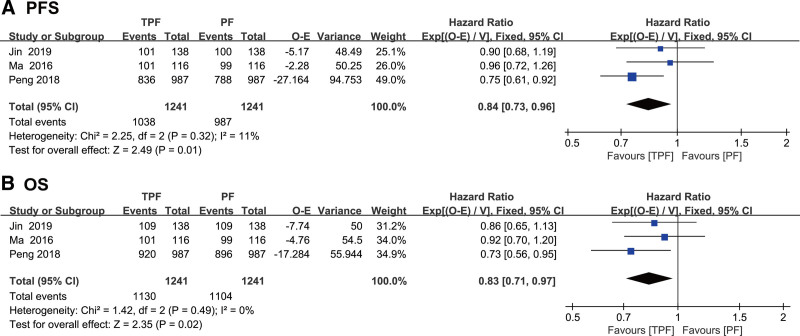
Forest plot for progression-free survival (PFS) and overall survival (OS) showing results comparing TPF plus CCRT against PF plus CCRT. (A) PFS; (B) OS. CCRT = concurrent chemoradiation, PF = cisplatin and fluorouracil, TPF = taxane, cisplatin and fluorouracil.

All 3 studies assessed PFS as the primary outcome. As shown in Figure 2A, the PFS in the TPF followed by CCRT arm was significantly improved compared with that in the PF followed by CCRT arm (HR 0.84 [95% CI 0.73–0.96], *P* = .01). There was no significant heterogeneity in PFS (I^2^ = 11%, *P* = .32). In terms of OS (Fig. 2B), there were statistically significant differences between the TPF followed by CCRT arm and PF followed by CCRT arm (HR 0.83 [95% CI 0.71–0.97], *P* = .02). No significant heterogeneity was observed in OS (I^2^ = 0%, *P* = .49).

### 3.4. DMFS and LRRFS

**Figure 3.**
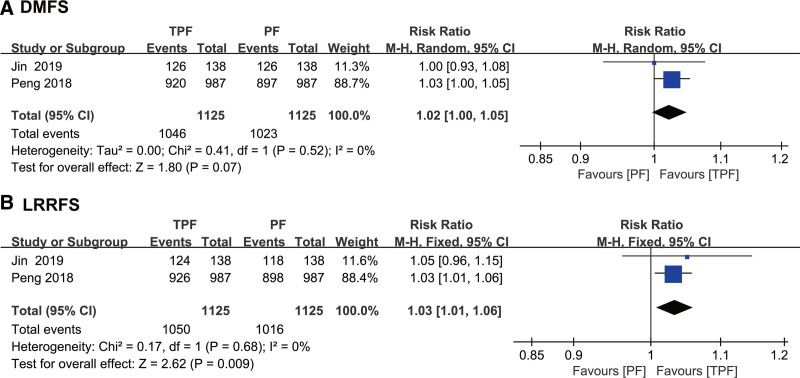
Forest plot for distant metastasis-free survival (DMFS), locoregional recurrence-free survival (LRRFS), showing results comparing TPF plus CCRT against PF plus CCRT. (A) 3-year DMFS; (B) 3-year LRRFS. CCRT = concurrent chemoradiation, PF = cisplatin and fluorouracil, TPF = taxane, cisplatin and fluorouracil.

There were 2 trials reporting DMFS and LRRFS. Regarding DMFS (Fig. 3A), TPF plus CCRT was not statistically superior to PF plus CCRT (RR 1.02 [95% CI 1.00–1.05], *P* = .07), and no significant heterogeneity was observed in 3-year DMFS (I^2^ = 0%, *P* = .41). In terms of LRRFS, as shown in Figure 3B, TPF plus CCRT was statistically superior to PF plus CCRT (RR 1.03 [95% CI 1.01–1.06], *P* = .009), and there was no heterogeneity in 3-year LRRFS (I^2^ = 0%, *P* = .68).

### 3.5. Severe toxicities

Ma’s study was excluded to further analyze the toxicities due to a lack of detailed information on grade 3 to 4 AEs. The incidences of grade 3 to 4 hematological toxicities and nonhematological toxicities during ICT were collected. As shown in Table 2, the most common grade 3 to 4 AEs during ICT included anemia, neutropenia, thrombocytopenia, nausea, vomiting, and diarrhea. We found that the incidence of neutropenia (RR 2.08 [95% CI 1.84–2.36], *P* < .001) and diarrhea (RR1.94 [95% CI 1.07–3.52], *P* = .03) were higher in the TPF plus CCRT group than in the PF plus CCRT group, while there were no significant differences between the 2 groups in the incidences of grade 3 to 4 AEs, such as anemia, thrombocytopenia, nausea, vomiting, hepatotoxicity and nephrotoxicity (all *P* > .05).

**Table 2:** Summary of the main grade 3 to 4 AEs during ICT in each clinical study.

Grade 3 or 4 AEs	Jin (2019) (events, %)		Peng (2018) (events, %)		TPF + CCRT versus PF + CCRT			
	TPF + CCRT (n = 138)	PF + CCRT (n = 138)	TPC + CCRT (n = 987)	PF + CCRT (n = 987)	RR [95% CI]	*P*	I^2^ (%)	*P*
Hematologic								
Anaemia	3 (2.2)	3 (2.2)	24 (2.4)	12 (1.2)	1.80 [0.96, 3.37]	.07	0	.4
Neutropenia	88 (63.8)	39 (28.3)	445 (45)	217 (22)	2.08 [1.84, 2.36]	<.001	0	.6
Thrombocytopenia	3 (2.2)	2 (1.4)	22 (2.2)	15 (1.5)	1.47 [0.80, 2.71]	.22	0	1
Non-hematologic								
Nausea	12 (8.7)	18 (13.0)	19 (1.9)	17 (1.7)	0.89 [0.55, 1.42]	.61	13	.3
Vomiting	6 (4.3)	14 (10.1)	51 (5.1)	55 (5.6)	0.83 [0.59, 1.16]	.27	57	.1
Diarrhoea	10 (7.2)	4 (2.9)	21 (2.1)	12 (1.2)	1.94 [1.07, 3.52]	.03	0	.6
Liver	0 (0)	0 (0)	21 (2.1)	13 (1.3)	1.62 [0.81, 3.21]	.17	–	–
Renal	0 (0)	0 (0)	1 (0.001)	0 (0)	3.00 [0.12, 73.55]	.5	–	–

AEs = adverse events, CCRT = concurrent chemoradiation, ICT = induction chemotherapy, PF = cisplatin and fluorouracil, RR = risk ratios, TPF = taxane, cisplatin and fluorouracil.

## 4. Discussion

ICT followed by CCRT has become the standard of care for LA-NPC.^[34]^ Recently, the most common ICT regimens for clinical practice included PF, TP, TPF, and GP, and phase III randomized trials have revealed that those ICT regimens plus CCRT improved the prognosis of patients with LA-NPC.^[35–37]^ However, it remains unclear which is the most effective ICT regimen with fewer side effects for LA-NPC. A Bayesian network meta-analysis investigated the efficacy and toxicity of 4 most common ICT regimens (TP, TPF, PF, and GP) followed by CCRT in LA-NPC, and the results indicated that there were no significant differences in OS and PFS among those 4 ICT regimens in LA-NPC.^[38]^ Nevertheless, another Bayesian network meta-analysis^[39]^ indicated that adding docetaxel might provide better efficacy than a regimen without docetaxel in OS, and TPF was the highest probability (TPF, TP, PF were 49.61%, 47.45%, and 1.57%, respectively) to be the optimal choice in LA-NPC, along with significantly increased incidence of worse AEs, especially in ≥ grade 3 hematological toxicity and oral mucositis. In 2021, an updated network meta-analysis^[40]^ revealed that TP- and GP-ICT regimens with better survival in LA-NPC than PF, MEPFL, and should be considered the new standard option. Because the optimal ICT regimens for LA-NPC are still controversial, we performed this meta-analysis to assess the efficacy and safety of the TPF-IC regimen plus CCRT versus the PF-IC regimen plus CCRT to determine the ideal regimen in LA-NPC. To our knowledge, this meta-analysis is the first study that includes “head-to-head” RCTs and retrospective PSM study to compare TPF or PF ICT regimens followed by IMRT concurrent with triweekly or weekly cisplatin.

In our study, we found that the TPF plus CCRT arm significantly improved PFS, OS, and LRRFS compared with the PF plus CCRT arm in LA-NPC. Similarly, Peng et al^[41]^ found that 3-year disease-free survival (76.2% vs 67.5%), LRRFS (92.0% vs 87.5%), OS (88.3% vs 84.1%), and DMFS (81.9% vs 75.0%; all *P* < .05) in TPF was superior to PF in the high-risk group, while survival outcomes did not significantly different in the low-risk group of stage III to IVa NPC. In consistently, a randomized phase II clinical trial^[42]^ on children and adolescents with stage IIB to IV NPC who received TPF or PF 3 weekly for 3 cycles of ICT followed by CCRT was conducted. The results indicated that the 3-year OS (85.7% vs 78.0%, *P* = .48) was not different between the 2 treatment arms. However, in the real world, Peng et al^[43]^ found that TPF plus CCRT and TP plus CCRT achieved significantly better 10-year OS than PF plus CCRT in both the whole cohort and the selected pairs by PSM analysis of patients with stage III to IVA NPC, this study was not included because the CCRT regimen included cisplatin or nedaplatin. Another retrospective study^[44]^ indicated that the TPF plus CCRT group had significantly improved 5-year OS (88.1% vs 80.7%; *P* = .042), DSS (88.5% vs 80.7%; *P* = .021), and DMFS (87.9% vs 78.6%; *P* = .012) rates compared with the PF plus CCRT group in LA-NPC, this study was not included due to 2D-CRT. Regarding DMFS, inconsistently, our results showed that TPF plus CCRT was not statistically superior to PF plus CCRT. Of note, patients with worse T and N stages are more prone to distant metastasis. Thus, personalized risk assessment to guide decisions about ICT regimens is needed.^[5]^

The safety profile of treatment is also a crucial issue. We also found that TPF plus CCRT significantly increased the incidence of grade 3 or 4 neutropenia compared with PF plus CCRT in LA-NPC. However, meta-analysis confirmed that with the established use of granulocyte-colony stimulating factor, the incidence of neutropenia has decreased substantially.^[45]^ Similarly, retrospective study^[43]^ reported that in the era of IMRT, PF followed by the CCRT group experienced significantly fewer grade 3 to 5 AEs than TPF followed by the CCRT group in LA-NPC. Conversely, Jan B et al^[46]^ revealed that TPF followed by the CCRT arm was better tolerated and the rates of death from toxic effects were lower than PF followed by the CCRT arm (2.3% vs 5.5%) in patients with stage III to IV unresectable squamous-cell carcinoma of the head and neck. Inconsistently, there was no difference between TPF followed by the CCRT arm and PF followed by the CCRT arm in terms of toxicity in children and adolescents with stage IIB to IV NPC.^[42]^

Based on previous studies, a higher dose of the TPF regimen will lead to more patients experiencing dose reduction and compliance reduction, along with more grade 3 to 5 AE toxicity. The completion rate of 3 cycles of CCRT is only approximately 30%, while the completion rate of 2 cycles of CCRT is more than 80%. Previous studies revealed that cumulative 200 mg/m² cisplatin may be the optimal dose in CCRT alone^[47]^ and 160 mg/m² cisplatin when receiving additional ICT to CCRT.^[48]^ Therefore, patients with LA-NPC received 2 to 3 cycles of the TPF regimen (docetaxel 60 mg/m^2^ plus cisplatin 60 mg/m^2^ plus 5 fluorouracil 600 mg/m^2^/day for 120 hours), and 2 concurrent cycles of cisplatin 80 or 100 mg/m^2^ may be an alternative treatment. However, in clinical practice, the most appropriate ICT regimen for individualized treatment must be determined based on clinical judgment, evaluation of the risk of local and distant relapse, and discussion the potential risks and benefits of the different treatments with the patient.

This present study has some limitations. First, the limitation is the number of included studies, and the analysis is based on published data and does not include individual patient data. In addition, the data all from the most endemic area may cause bias to the largest population, there are few data on late toxicity compared with acute toxicity and Ma’s study lacks detailed toxicity profiles. Furthermore, a comprehensive literature database and manual searches were conducted, but we were not able to obtain additional unpublished data. Overall, the quality of the studies included in this review can be considered high.

## 5. Conclusions

Our study indicated that the TPF induction regimen followed by the CCRT arm is superior to the PF plus CCRT arm in LA-NPC in the treatment efficacy of OS, PFS, LRRFS, and along with higher risk of grade 3 to 4 neutropenia and diarrhea. However, these effects resolve with preventive and interventional treatments. Thus, TPF plus CCRT appears to be a reasonable treatment option in the IMRT era in LA-NPC. To validate our conclusion, more “head-to-head” prospective and large sample studies to compare different ICT regimens plus CCRT are needed.

## Acknowledgments

We thank all authors whose publications could be adopted in our meta-analysis.

## Author contributions

**Conceptualization:** Lili Yu.

**Data curation:** Haiwen Li.

**Formal analysis:** Qibiao Wu.

**Funding acquisition:** Wenmei Su.

**Methodology:** Qibiao Wu, Jiayuan Wu.

**Supervision:** Wenmei Su, Lili Yu.

**Writing – original draft:** Haiwen Li, Haiqing Luo.

**Writing – review & editing:** Lili Yu.
